# Reactive Arthritis or HIV-Associated Arthropathy: Is It Important to Differentiate Them?

**DOI:** 10.7759/cureus.69788

**Published:** 2024-09-20

**Authors:** Juan Camilo Santacruz, Marta Juliana Mantilla, Sandra Pulido, Carlos Alberto Agudelo, Juan Diego Londoño, John Londono

**Affiliations:** 1 Spondyloarthropathies Research Group, Universidad de La Sabana, Chía, COL; 2 Rheumatology Department, Centro de Investigación en Reumatología y Especialidades Médicas (CIREEM), Bogotá, COL; 3 Rheumatology Department, Clínica las Américas Auna, Medellín, COL; 4 Rheumatology Department, Universidad Nacional de Colombia, Bogotá, COL

**Keywords:** hiv-associated arthropathy, hla-b27, reactive arthritis, treatment choices, tumor necrosis factor inhibitors

## Abstract

Although human immunodeficiency virus (HIV)-associated arthropathy is the most frequently described joint syndrome, the spectrum of its clinical manifestations is poorly known, and it is difficult to distinguish it from reactive arthritis (ReA). Knowing how to differentiate these two conditions has major implications regarding their prognosis and treatment. We present the case of an adult patient with a history of HIV infection with adequate virological control and good adherence to antiretroviral treatment, which began with an acute clinical picture consisting of additive asymmetric oligoarthritis with subsequent transition to symmetric polyarthritis predominantly in the upper extremities, initially attributed to ReA. Finally, his immunoserological profile was determined with negative results for rheumatoid factor, anti-citrulline antibodies, and human leukocyte antigen B27, achieving complete resolution of joint symptoms five weeks after treatment with nonsteroidal anti-inflammatory drugs, hydroxychloroquine, and intermediate doses of glucocorticoids, establishing the diagnosis of HIV-associated arthropathy.

## Introduction

Musculoskeletal manifestations associated with human immunodeficiency virus (HIV) have been described since the beginning of its global epidemic. The first reports of the symptoms related to rheumatic diseases were described three years after its discovery, with Winchester et al. describing the first case of reactive arthritis (ReA) in a patient with acquired immunodeficiency syndrome (AIDS) [1]. Patients who are HIV positive manage to have a longer survival in the era of antiretroviral therapy, making chronic non-communicable diseases such as rheumatic diseases become an important cause of morbidity, greatly affecting their quality of life. Joint syndromes that have been described as associated with HIV include HIV-associated arthropathy, rheumatoid arthritis, seronegative spondyloarthritis (ReA, psoriatic arthritis, and undifferentiated spondyloarthritis), painful joint syndrome, and diffuse infiltrative lymphocytosis syndrome (the latter most frequently described in HLA-DRB1-positive African Americans before highly active antiretroviral therapy) [2]. The pathophysiological mechanisms leading to the development of rheumatic manifestations in HIV infection remain unclear but appear to be multifactorial. Joint damage by direct viral infection, polyclonal activation of B cells, and genetic and environmental factors have been proposed [3]. ReA in HIV patients has been widely described, and it has been suggested that the pathogenesis is related to human leukocyte antigen B27 (HLA-B27) positivity, this being the main susceptibility factor. The association between ReA and AIDS can be explained in some way by immunosuppression that predisposes to invasion by arthritogenic microorganisms [4]. In addition, patients with AIDS present a wide range of genitourinary and gastrointestinal infections (*Cryptosporidium* sp., *Isospora belli*, *Candida* sp., *Shigella* sp., *Entamoeba histolytica*, *Giardia lamblia*, *Chlamydia trachomatis*, and *Cytomegalovirus*). Some of these proposed microorganisms have been related to the pathogenesis of ReA and generally tend to be asymptomatic in HIV-positive patients, although other studies suggest that the virus itself has a direct involvement in the genesis of the disease [5,6]. In routine clinical practice, it is difficult to distinguish between HIV-associated arthropathy (which is the most common entity) and ReA, even more so if the HLA-B27 result is negative. The lack of differentiation between these two conditions has important prognostic implications since HIV-associated arthropathy tends to have a more acute polyarticular involvement and sometimes requires the intensification of immunosuppressive treatment unnecessarily given that it tends to have a self-limiting behavior. Here, we present a case of a patient with HIV-associated arthropathy with partial response to nonsteroidal anti-inflammatory drugs (NSAIDs), achieving resolution of joint symptoms after the administration of hydroxychloroquine and glucocorticoids at intermediate doses.

## Case presentation

**Figure 1:**
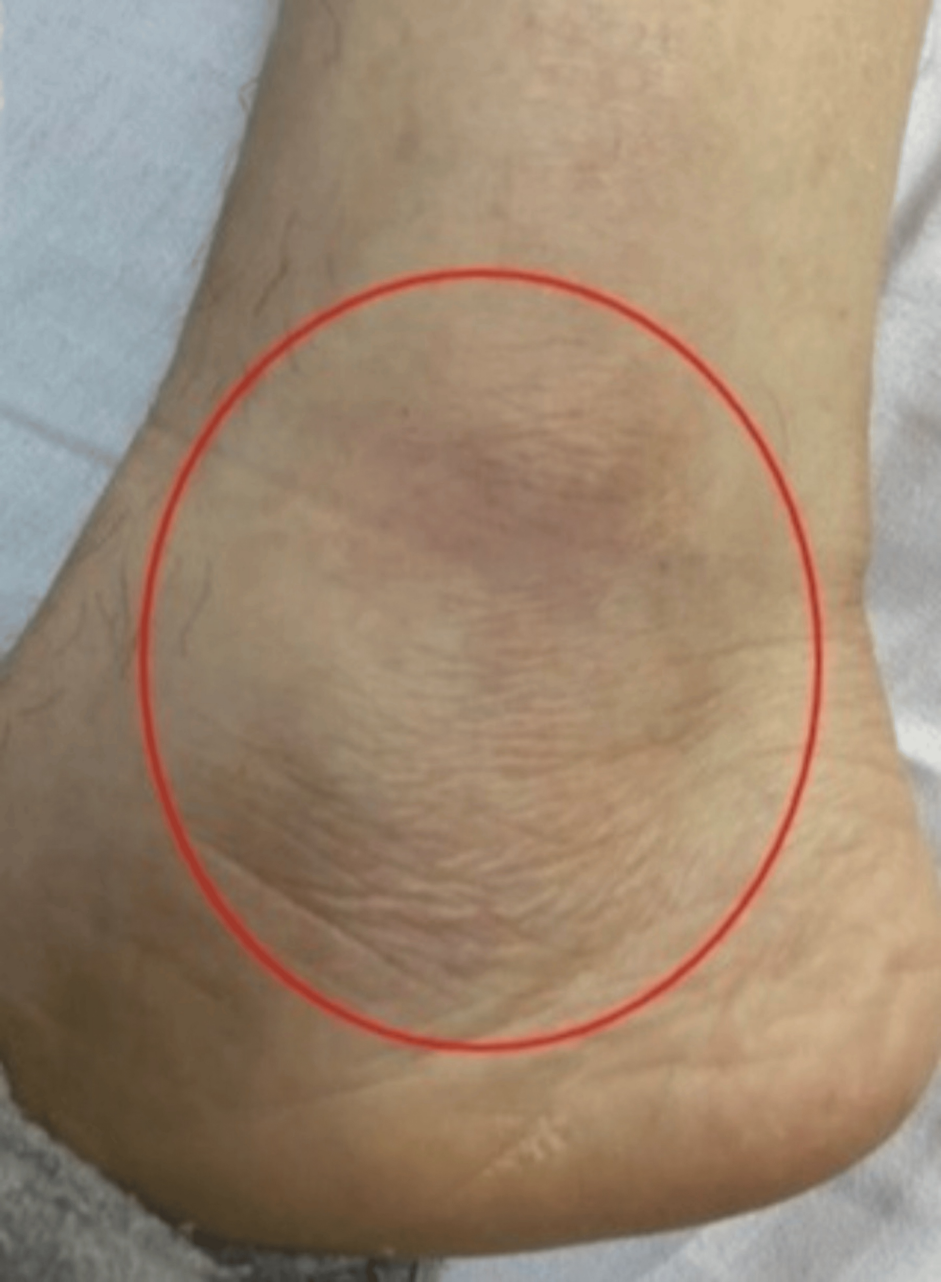
Left ankle synovitis

**Figure 2:**
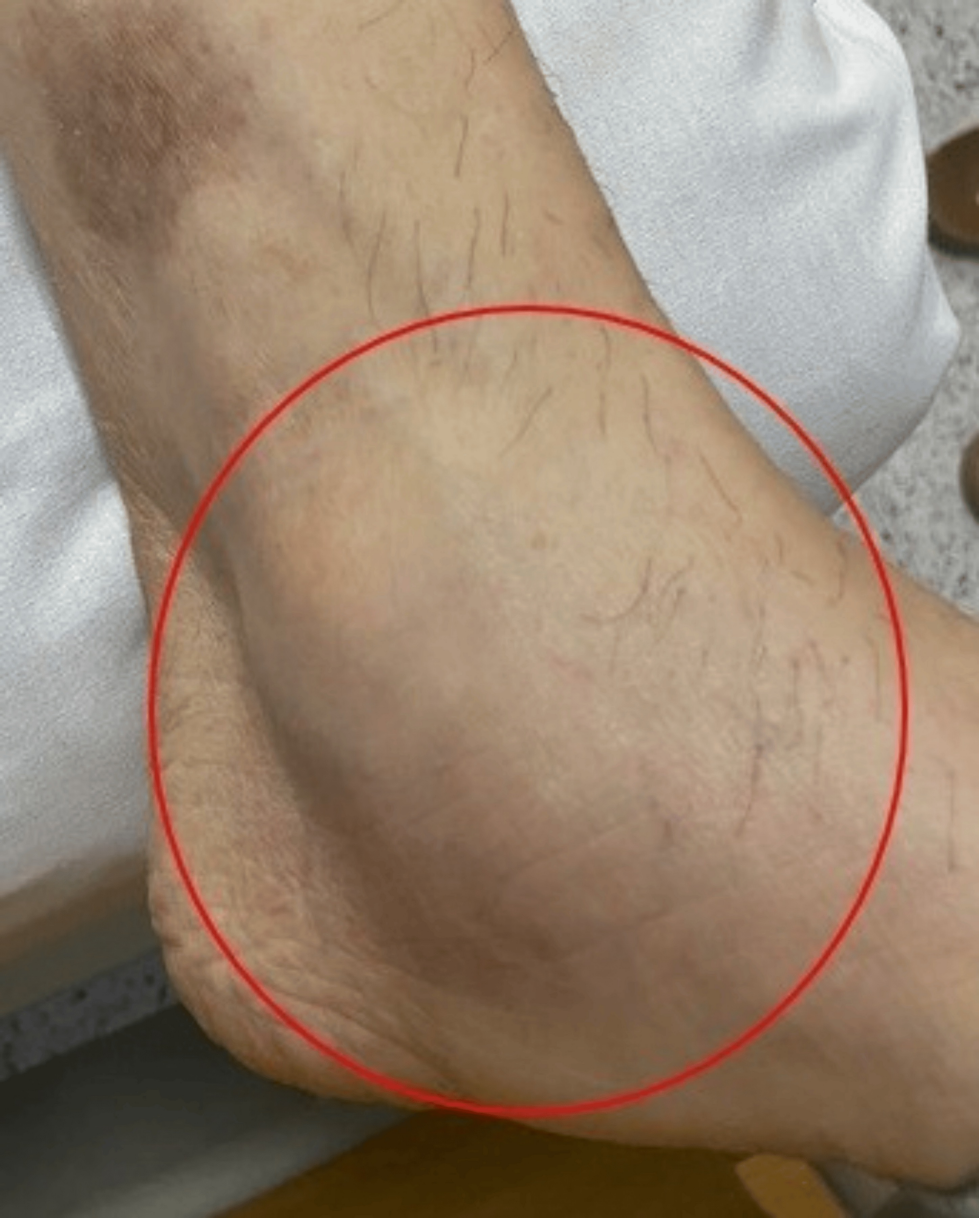
Right ankle synovitis There is marked synovitis of the right ankle highlighted by a red circle that delimits the area of ​​edema.

**Figure 3:**
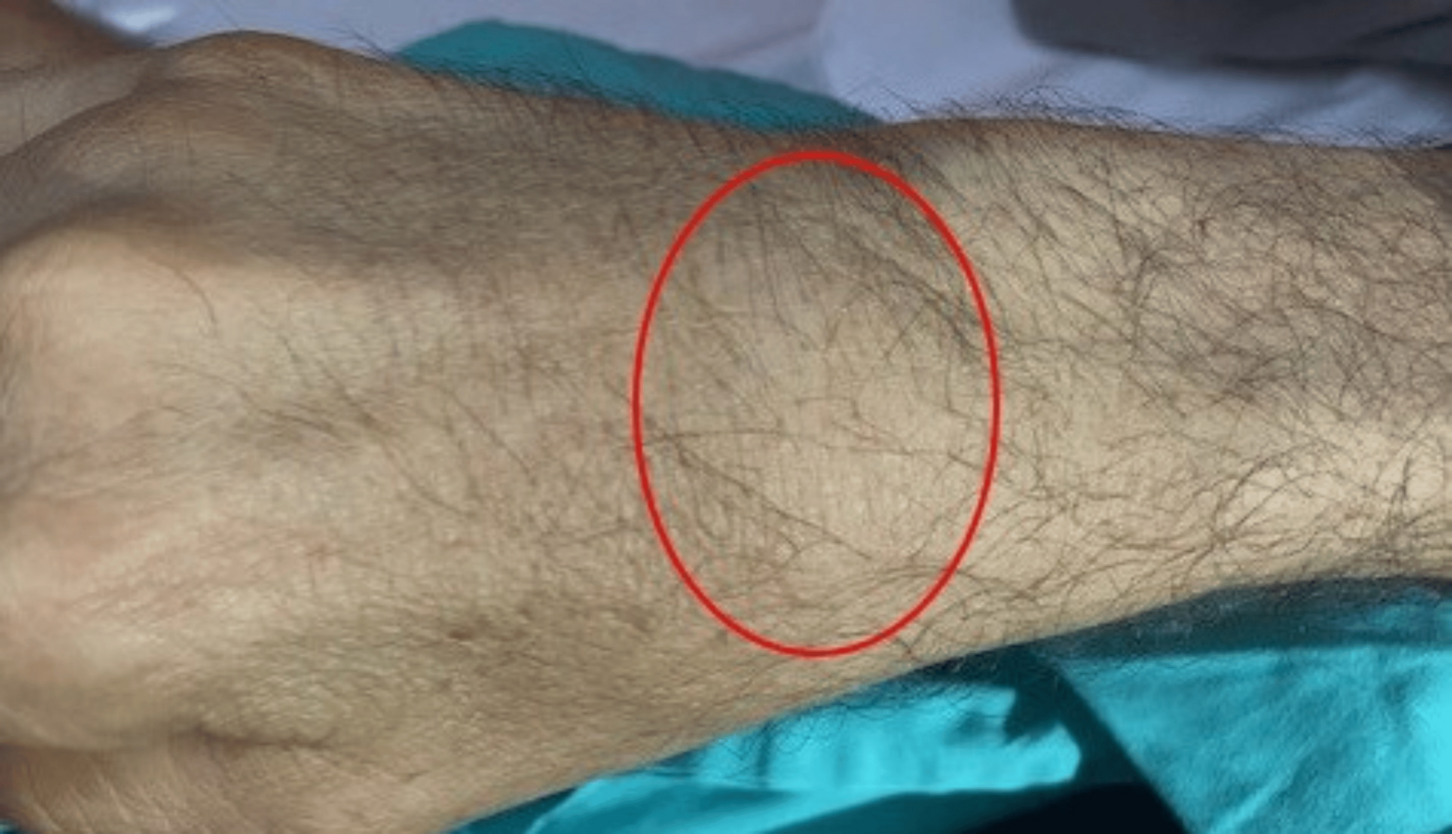
Right carpal synovitis

A 27-year-old male patient was admitted to the emergency department due to a five-day clinical history of asymmetric, additive oligoarticular pain, predominantly in the upper extremities (right elbow and carpus), with subsequent change to polyarticular involvement (two days later) due to the presence of synovitis in the ankles and knees in a symmetrical manner. The joint pain was continuous, causing limitation of movement mainly in the upper extremities, with no improvement after the administration of 75 mg of diclofenac and 8 mg of dexamethasone intramuscularly. Medical history includes HIV infection on antiretroviral therapy (darunavir/ritonavir 100 mg/day, lamivudine/abacavir 300/600 mg/day) with the latest undetectable viral load and CD4 count of 364 cells/mm3, along with a report of non-reactive VDRL and FTA-ABS, non-reactive hepatitis C antibodies, and antibodies against hepatitis B surface antigen greater than 1000 IU/ml reported in the last outpatient infectious disease assessment performed two months ago. The review of systems did not report any enteric, respiratory, or genitourinary infection before the presentation of joint pain and did not mention any extra-articular manifestation of ReA (ocular symptoms, oral ulcers, skin lesions, or nail changes). The physical examination on admission confirmed the presence of synovitis of the right elbow and bilateral carpal joints, along with bilateral suprapatellar and tibiotalar synovitis (Figures 1-3).

Magnetic resonance imaging (MRI) of the upper and lower extremities was performed, describing changes in synovitis of the right elbow without evidence of subchondral involvement, changes in bilateral tibiotalar synovitis without evidence of erosions, flexor tenosynovitis, and a ruptured Baker's cyst identified on ultrasound of the right knee, with the conservative treatment suggested by orthopedics. He was initially evaluated by rheumatology considering the diagnosis of HIV-associated ReA indicating a single intravenous (IV) pulse of methylprednisolone 250 mg, extending the administration of NSAIDs (diclofenac 75 mg IV daily for five more days), the start of hydroxychloroquine 200 mg daily, and low doses of prednisolone (5 mg daily). The patient presented with this intervention an improvement of the pain and edema of the affected joint groups, to a greater extent associated with the administration of glucocorticoids. Later, he presented a slight elevation of transaminases, so he was evaluated by hepatology considering the possibility of acute liver injury associated with NSAIDs, indicating that they should be discontinued and requiring an increase in the prednisolone dose to 15 mg daily. Finally, the patient achieved complete resolution of synovitis with this dose in five weeks, completing his evaluation with the result of negative rheumatoid factor, negative anti-citrulline antibodies, and negative HLA-B27. An additional study of synovial fluid was performed, but due to the presence of a clot, it was not possible to perform the leukocyte and erythrocyte count. With all the results of the complementary tests and the recovery time, the diagnosis was finally reclassified as HIV-associated arthropathy. Table 1 describes the most representative laboratory studies on admission and before hospital discharge.

**Table 1:** Laboratory parameters upon admission to hospital and prior to discharge ALT: alanine transaminase, AST: aspartate aminotransferase, ANAS: antinuclear antibodies, ENAS: extractable nuclear antigens, HLA: human leukocyte antigen

Paraclinical studies	Upon admission	Before discharge
Hemoglobin (gr/dL)	13.4	11.5
Hematocrit	39.1	33.2
Mean corpuscular volume (fL)	85.3	80.3
Total leukocyte count (/µl)	18660	10410
Total lymphocyte count (/µl)	2010	2523
Total platelet count (/µL)	362000	409000
Creatinine (mg/dL)	0.85	0.7
Urea Nitrogen (mg/dL)	22	18
Erythrocyte sedimentation rate (mm/h)	120	-
C-reactive protein (mg/dL)	23.5	12
ALT U/L	40	147
AST U/L	42	79
ANAS (titers)	Hep 2 positive 1/160 (fine granular pattern) AC-01	-
ENAS (U/mL)	Negatives	-
Anti-DNA (UI/mL)	Negative	-
C3 (mg/dL)	176	-
C4 (mg/dL)	38	-
Rheumatoid factor (U/ml)	12 (negative for cut-off 14UI/ml)	-
Anticitrulline antibodies (U/ml)	Less than 0.8 (negative)	-
HLA-B27	Negative	-
Urethral discharge culture (Thayer Martin)	Negative	-
Urine and blood cultures	Negative	-
Glucose (mg/dL)	127	-
Urinalysis	Negative for infection - amorphous urate crystals	Normal

## Discussion

HIV-associated arthropathy tends to present as asymmetric oligoarthritis, symmetric polyarthritis, or monoarthritis. The asymmetric oligoarthritis presentation is the most common form, tends to be male-predominant, and primarily involves the knees and ankles [7]. When it presents as the symmetric polyarthritis variant, it largely mimics rheumatoid arthritis, even presenting with similar deformities such as ulnar deviation. However, it tends to have a more acute onset at presentation and is usually non-erosive. Jaccoud arthropathy has also been seen within the spectrum of clinical manifestations associated with HIV arthropathy [8]. This entity tends to be short-lived, with peak intensity occurring between one and six weeks. Some patients may develop a destructive arthropathy with marked functional disability. Mucocutaneous and enthesis involvement is rare. Radiological changes may be similar to those of patients with rheumatoid arthritis, such as decreased joint space, erosions, and juxta-articular osteopenia, although some patients may present with new bone formation [9-11]. Antinuclear antibodies, HLA-27, and rheumatoid factor tend to be negative, supporting that HIV has a direct inflammatory effect on synovial tissue since P24 antigen, viral deoxyribonucleic acid, and tubuloreticular inclusions have been found in the synovial fluid of affected joints [12]. In cases of HIV-associated ReA, the characteristic clinical presentation is asymmetric oligoarthritis that most frequently affects the lower limbs and is usually accompanied by enthesitis. It is common to see some extra-articular cutaneous manifestations such as circinate balanitis (circinate vulvitis in women) and keratoderma blennorrhagica. In addition, these patients may present extensive symmetrical scaly plaques that are sometimes indistinguishable from psoriatic arthritis [13]. Urethritis presents with a similar frequency to that of patients with ReA without HIV. The involvement of other domains, such as uveitis and the axial axis, tends to be an unusual form of presentation. The distribution of HLA-B27 positivity is very heterogeneous around the world, being very prevalent in Caucasian populations (80-90%) as opposed to people of African descent, where its negativity is almost absolute. A lower frequency of involvement of the joints of the hand and wrists and multidigital dactylitis has been observed [14]. An important difference is that joint symptoms in HIV-associated arthropathy can manifest at any stage of the infection, while in ReA, they usually occur in advanced stages of the disease, particularly when AIDS is established [15]. Although there is no clear difference in the treatment of both entities, there has always been a concern about the loss of virological control after the use of immunosuppressive treatment. The suggested first-line treatment is NSAIDs at maximum anti-inflammatory doses [16]. The second line of treatment proposed is systemic glucocorticoids and disease-modifying antirheumatic drugs (DMARDs), especially in refractory and persistent cases. Among DMARDs, hydroxychloroquine has been the most effective and safe drug given its antiretroviral properties demonstrated in vitro. Sulfasalazine and cyclosporine have been proposed as alternatives, having a similar safety and efficacy profile [17]. Methotrexate (15 mg weekly) may also be considered as an alternative, even in patients with AIDS if chemoprophylaxis is initiated to prevent *Pneumocystis jirovecii* infection [18]. The use of tumor necrosis factor inhibitors (TNFi) has been a topic of discussion in the context of patients with ReA and HIV, although it has been suggested that it may be safe in patients who do not have AIDS and have been refractory to conventional DMARDs. In a study using TNFi, antimetabolites, and checkpoint inhibitors, where 95% of patients received concomitant antiretroviral therapy, there was a change in viral load (from undetectable to detectable) in the first year of treatment in 41.2%. In addition, 11.8% of patients presented an additional serious infection not directly associated with the drug, even with a mean CD4+ T cell count of 609 cells/μL. Given these results, careful monitoring is necessary to detect loss of virological control and incidences of infections [19]. There are few studies to evaluate the safety of TNFi in the context of HIV infection and associated autoimmune diseases. One of the most representative was the one conducted by Wangsiricharoen et al., who conducted a study to evaluate the incidence of serious infections in patients with HIV and previous therapy with TNFi for the treatment of autoimmune diseases between January 1999 and March 2015. Twenty-three patients met the inclusion criteria, and of these 16 were treated with etanercept (dose and duration not specified), six with adalimumab, and four with infliximab. In total, two patients (8.7%) reported at least one serious infection (*Streptococcus pyogenes* pneumonia with empyema and methicillin-sensitive *Staphylococcus aureus* infection), and both were receiving treatment with etanercept monotherapy or co-therapy with etanercept, sulfasalazine, and prednisone, respectively. The incidence rate per 100 patient-years was 3.28 among patients with a viral load greater than 500/ml at the start of treatment and 2.09 in patients with a viral load less than 500/ml, being generally low without significant differences even in those who did not have stricter virological control. It should be noted that only 4.3% of patients in this study had ReA as the main diagnosis [20].

## Conclusions

HIV-associated arthropathy may have a more acute clinical course, with a presentation of asymmetric oligoarthritis, polyarthritis, or HLA-B27-negative monoarthritis with the absence of enthesitis, dactylitis, or extra-articular manifestations that are more characteristic of ReA. It is necessary to wait for six weeks to clarify the diagnosis and, therefore, the prognosis while waiting to define whether it is self-limiting or if more clinical manifestations of ReA are supported (the latter does not tend to debut as mono- or polyarthritis). The treatment of both entities is similar, although clarifying that it is an HIV-associated arthropathy would suggest initially giving measures to control joint symptoms with NSAIDs or glucocorticoids in the acute phase given the low probability of progressing to a chronic phase before considering the early initiation of TNFi. An important difference is that joint symptoms in HIV-associated arthropathy can manifest at any stage of the infection, whereas in ReA they usually occur in advanced stages of the disease. Further studies are required to assess the safety of TNFi in this context.
